# Sociodemographic factors as a predictor for pregnancy-related anxiety

**DOI:** 10.1192/j.eurpsy.2024.670

**Published:** 2024-08-27

**Authors:** M. Abdelkefi, R. Walha, R. Feki, W. Zid, I. Gassara, N. Smaoui, S. Omri, N. Charfi, L. Zouari, J. Ben Thabet, M. Maalej Bouali, K. Chaabene, M. Maalej

**Affiliations:** ^1^psychiatry C department; ^2^Gynecology-Obstetrics department, Hedi Chaker university hospital, sfax, Tunisia

## Abstract

**Introduction:**

Pregnant women are particularly vulnerable to a wide variety of psychiatric symptoms, including anxiety related to pregnancy and childbirth.

**Objectives:**

The purpose of our study was to determine the sociodemographic characteristics of pregnant women and investigate their relationship with pregnancy-related anxiety.

**Methods:**

The study was conducted from February to July 2023 among pregnant women in their 3rd-trimester consulting at the Gynecology-obstetrics department of the Hedi Chaker University Hospital of Sfax, Tunisia. Women with obstetric conditions favorable to vaginal delivery (cephalic presentation and eutrophic fetus) were interviewed using a questionnaire including their sociodemographic characteristics and the brief version of the pregnancy-related anxiety questionnaire PRAQ-R2.

**Results:**

A total of 350 women were included in our study. The mean age of the participants was 28 years [16-41 years] with the majority being married (95.7%). One hundred and eighty-eight women (53.7%) did not graduate from high school and 213 (60.9%) were housewives. Half of the participants (52.9%) lived in the city, and 38.9% reported low income. Almost half of them (46.28%) were multiparous.

The mean score of the PRAQ-R2 was 31.24 ± 7.53.

We found a positive correlation between the PRAQ-R2 scale score and age younger than 30 years (p<0.001), low educational level (p=0.006), and low income (p=0.031).

**Conclusions:**

Our findings suggest that demographic factors seem to predict anxiety related to pregnancy and are worth examining in future studies for a better understanding of this symptom in pregnant women.

**Disclosure of Interest:**

None Declared

